# Addressing the plasmonic hotspot region by site-specific functionalization of nanostructures†

**DOI:** 10.1039/c9na00757a

**Published:** 2019-12-04

**Authors:** Eric S. A. Goerlitzer, Lutz E. Speichermann, Talha A. Mirza, Reza Mohammadi, Nicolas Vogel

**Affiliations:** Institute of Particle Technology (LFG), Friedrich-Alexander University Erlangen-Nürnberg (FAU) Cauerstraße 4 D-91058 Erlangen Germany nicolas.vogel@fau.de; Institute of Nanotechnology (INT), Karlsruhe Institute of Technology (KIT) Hermann-von-Helmholtz-Platz 1 76344 Eggenstein-Leopoldshafen Germany; Interdisciplinary Center for Functional Particle Systems (FPS), Friedrich-Alexander University Erlangen-Nürnberg (FAU) Haberstraße 9 D-91058 Erlangen Germany

## Abstract

Strong electromagnetic fields emerge around resonant plasmonic nanostructures, focusing the light in tiny volumes, usually referred to as hotspots. These hotspots are the key regions governing plasmonic applications since they strongly enhance properties, signals or energies arising from the interaction with light. For a maximum efficiency, target molecules or objects would be exclusively placed within hotspot regions. Here, we propose a reliable, universal and high-throughput method for the site-specific functionalization of hotspot regions over macroscopic areas. We demonstrate the feasibility of the approach using crescent-shaped nanostructures, which can be fabricated using colloidal lithography. These structures feature polarization-dependent resonances and near-field enhancement at their tips, which we use as target regions for the site-selective functionalization. We modify the fabrication process and introduce a defined passivation layer covering the central parts of the gold nanocrescent, which, in turn, selectively uncovers the tips and thus enables a localized functionalization with thiol molecules. We demonstrate and visualize a successful targeting of the hotspot regions by binding small gold nanoparticles and show a targeting efficiency of 90%. Finally, we demonstrate the versatility of the method exemplarily by translating the principle to different nanostructure geometries and architectures.

## Abstract 2

We provide a simple and parallel method to selectively functionalize the hot-spot regions of plasmonic nanostructures, allowing to deposit molecules or particles directly at the most active sites.

## Graphical abstract

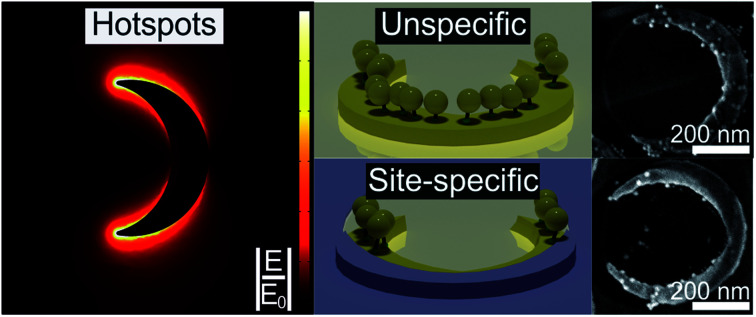

## Introduction

Electromagnetic radiation impinging on noble metal nanostructures excites collective oscillations of the free electron gas, which are known as localized surface plasmon resonances (LSPR). Along with such resonant behavior, strong electrical fields emerge around these structures, focusing the light into tiny volumes. These volumes are usually referred to as optical hot-spots, as the electromagnetic field can reach values magnitudes higher than the incoming light.^1–4^

Such hotspots can be useful to enhance properties arising from the interaction of radiation with the nanostructure and the surrounding environment and are therefore applied, for example, to increase the local temperature,^5^ catalyze chemical reactions,^4,6–8^ increase photovoltaic efficiency,^9^ or increase spectroscopic signals.^10,11^ The latter has been particularly useful in surface-enhanced Raman spectroscopy (SERS),^4^ where optimized design of such hotspots has shown to be capable of the detection on the single-molecule level.^12,13^ Two very recent reviews highlighted the use of plasmonic hot spots in chemical transformations and SERS, while underlining the demand for reliable methods for substrate fabrication.^4,8^

An ideal nanostructure to take advantage of signal, energetic, or chemical enhancement should have the following properties. First, its structure should provide a high near-field enhancement. Second, these near-fields should be confined to a small volume at the nanostructure's surface. Third, the targeted molecules or objects should be selectively directed to these hotspots. Fourth, the plasmonic substrates need to allow reproducibility and uniformity over time and space.^4^

The first two points can be satisfied either with complex anisotropic noble-metal nanostructures that feature sharp tips,^1–3^ or by placing nanostructures in close proximity to induce coupling between the individual resonances.^14–16^ However, not all positions on the surface of a nanostructure are equally efficient to harvest plasmonic properties. Ideally, the targeted molecules would be exclusively placed within hotspot regions of high near-field intensity, instead of being evenly distributed throughout the surface of the nanostructure or substrate. Such a controlled placement would increase the sensitivity in sensing applications, since only the most active part of the surface is used for the signal enhancing. Additionally, a selective functionalization of the hotspots could allow binding of additional plasmonic particles onto the hotspot region, thus further enhancing the electromagnetic near-field *via* coupling of plasmonic resonators.

Thiol chemistry presents a convenient way to selectively functionalize the entire exposed noble metal surface, allowing a localized control of the surface chemistry and thus introduce specific binding motifs able to selectively anchor molecules or particles onto a noble metal nanostructure.^17,18^ However, this leads to a homogenous coverage of all the regions of the gold structure.

One possibility to achieve a localized functionalization of hot-spot regions is to exploit the very nature of the near fields itself to selectively control chemical binding with the help of near fields of plasmonic modes. This was previously achieved by using bright and dark plasmonic modes that were matching the spectral properties required for the photochemical reactions.^19–23^ While these approaches show the potential, they require a high level of sophistication since the near-field itself provides the structuring, which, besides the spectral matching, requires also illumination optimization and timing during the functionalization.

**Fig. 1:**
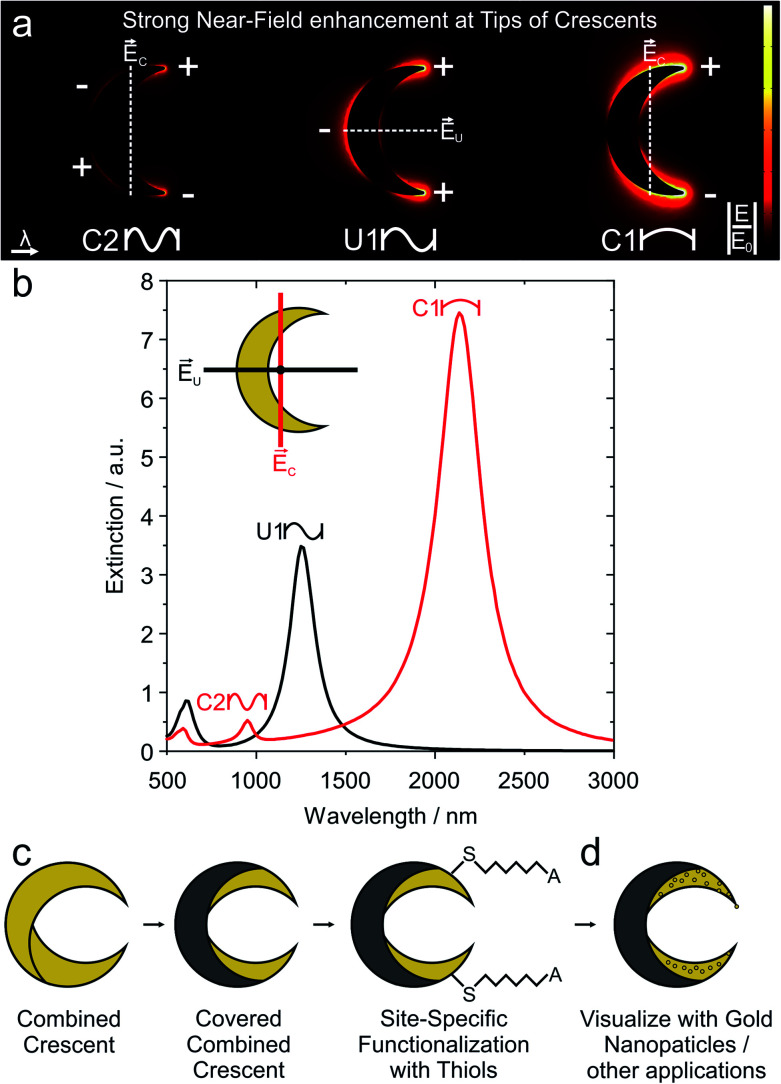
Strategy for site-specific functionalization of the hotspot regions of gold nanocrescents. (a) Nanocrescents are analogues of split-ring resonators and can evoke the fundamental dipole resonance (C1), the first order resonance (U1) and the second order resonance (C2). A common feature for all of these resonances is the enhanced electric field (hot spot) around their tips. (b) A typical extinction spectra is given for the two polarizations. For the C polarization, the electric field is parallel to the long axis of a crescent, which reads as a C. In 90° tilted case the electric field is along the short axis of a crescent, which now reads as a U. (c) Strategy to selectively functionalize the hotspot region at the tip. Conventional functionalization chemistry would address the entire surface of the gold nanostructure. First, a combined crescent with extended contour is produced by two gold deposition steps. Subsequently, the nanocrescent can be partly covered by evaporating a blocking layer. Finally, the structure can be functionalized with thiols, which exclusively bind to the free gold surface of the nanostructure. (d) To visualize the successful site-specific functionalization, we electrostatically adsorb gold nanoparticles, so that they can be seen in conventional electron microscopes.

A conceptually simpler strategy to achieve such a localized functionalization would be to manipulate the fabrication process directly to selectively expose certain regions of the nanostructures that show high electromagnetic fields. We employ the established fabrication method of nanocrescents^24–26^ as analogues of split-ring resonators,^27–29^ with polarization-dependent resonances,^24,30^ that can be controlled over a large spectral range,^25^ using various materials such as silver, gold or aluminum.^31–34^ Such nanocrescents are attractive structures to demonstrate our concept as they provide sharp tips with high near-field enhancements (Fig. 1a) with a simple fabrication process,^35,36^ and as they have been successfully employed as substrates for dielectric sensing,^35,37,38^ SERS,^39,40^ and to enable concentration measurements in microfluidics.^41^

In this manuscript, we modify the established fabrication process for gold nanocrescents to create a material contrast at the surface of the nanostructures. In this new process, only the hot-spot region of the gold nanocrescent remains uncovered and can subsequently be functionalized by thiol chemistry. This strategy allows direct access to hot-spot regions for applications that require optical and chemical hotspots,^8^ without the need for sophisticated photochemical patterning. We start with the extended gold nanocrescent, which is fabricated by a double deposition process and therefore termed combined nanocrescent.^25^ Subsequently, we deposit a passivation layer on the central part of the combined nanocrescent to selectively uncover the tip regions that feature the highest near-field enhancement (Fig. 1c). Next, the uncovered tips are selectively functionalized with thiols. We demonstrate that we can selectively address these regions by binding smaller gold nanoparticles at the tips of the underlying nanocrescent structure (Fig. 1d). This gold nanoparticle absorption allows us to visualize the thiol functionalization with conventional scanning electron microscopy.

Finally, we translate the principle to different geometries and architectures to demonstrate the flexibility of the approach that will allow substrate production in a wide range of applications.

## Results and discussion

### Fabrication of site-specific functionalized arrays of crescents

We used two methods to obtain colloidal masks. The first, well-established method builds on the deposition of a hexagonal close packed monolayer of polystyrene particles onto a glass substrate.^42^ To allow the desired deposition of material underneath the colloids, we reduced their size by exposing them to an oxygen plasma.^42^ The plasma etching provides flexibility, as the particle size can be controlled by the duration of oxygen plasma exposure. Yet, minute fluctuations occur, translating into a change in gold nanocrescent dimensions and thus a shift in the optical spectra, making it difficult to compare the spectra of different samples. This issue was overcome by a second method building on the deposition of core–shell particles,^43^ which, after removal of the soft-shell produce a hexagonal non-close packed array of inorganic core particles with a defined size. The latter method was used when the optical properties of different samples were systematically compared, while the former method provided an experimentally simpler access to the desired nanostructures for the subsequent functionalization experiments.

**Fig. 2:**
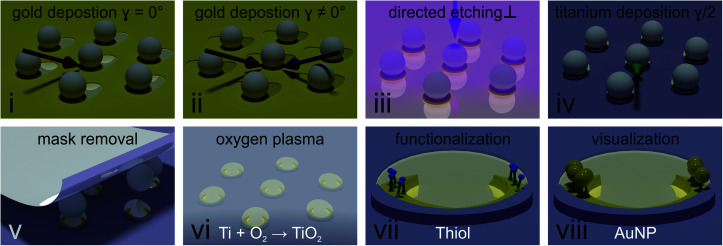
Fabrication of arrays of site-specific functionalized nanocrescents. (i) A thin layer of titanium (1 nm, as adhesion promoter) and gold (20 nm) are evaporated at an angle of 30° with respect to the surface normal (red arrow) to deposit gold underneath the masking particles. (ii) A second layer of gold is deposited at a different azimuthal angle *γ*, indicated by the blue arrow in the cartoon. This angle defines the overlap of the gold layers underneath the particle, and by this increases the contour of the crescent. (iii) Reactive ion etching along the surface normal (pink arrow) is used to remove material not covered by the shadow of the particle. (iv) Evaporation of a thin layer of titanium (5 nm) at half the previous azimuthal angle (*γ*/2) to passivate the central area of the crescent. The deposition of titanium with an azimuthal angle in between the two gold azimuthal angles (green arrow) ensures that the tips of the crescent remain uncovered. (v) Removal of the particle mask using adhesive tape. (vi) Oxidation of titanium to titania and removal of residuals of the particle mask by oxygen plasma. (vii) Selective functionalization of the uncovered region using thiol chemistry. (viii) Site-selective deposition by electrostatic attraction of negatively charged gold nanoparticles onto the tip region functionalized with positively charged cysteamine molecules.

Fig. 2 describes the process to selectively functionalize gold nanocrescent at their tip region in detail. (i) First, a thin layer of titanium (1 nm) used as adhesion promoter and gold (20 nm) is evaporated under an angle of 30° with respect to the surface normal (also see Fig. S1†) to deposit material underneath the colloidal particles acting as mask. (ii) In a second gold evaporation step, the sample is rotated in plane by the desired azimuthal angle *γ* (also see Fig. S1†). This rotation increases the area covered with gold underneath the particle mask and will increase the contour length of the combined crescent by the chosen angle *γ*. (iii) Directed dry etching with argon ions along the surface normal removes material not covered by the particles' shadows, producing the combined crescents. (iv) A thin layer of titanium (5 nm) is deposited at half the azimuthal angle (*γ*/2). This deposition in between the angles chosen for the gold evaporation ensures that the center of the combined crescents is covered by the titanium layer, while the tip regions remains uncovered. We choose titanium due to its affinity to oxidize into a dielectric passivation layer and its inertness to thiols. Similar results have been achieved using reactive evaporation of silicon monoxide, which forms a silicon dioxide passivation layer. (v) After removal of the particle mask with adhesive tape, undesired residuals were removed by cleaning with organic solvents, followed by an isotropic oxygen plasma treatment (vi) which oxidizes the titanium layer into dielectric titania. (vii) The material contrast between crescent center and tip region was used to selectively functionalize the tip region using thiol chemistry. We chose cysteamine to introduce positive charges, as the functional ammonia group (p*K*_A_ = 10.75), is positively charged in water at chosen pH = ∼4 (viii).

We validated the site-selective functionalization by the electrostatic adsorption of negatively charged, mercaptohexanoic acid-functionalized gold nanoparticles.

The chosen methodology affords several degrees of freedom to tailor the geometry and lattice of arrays of crescents. The crescent size is directly controlled by the diameter of the colloidal particles used as masks.^24,25,44,45^ The evaporation angle controls the width of the crescent (or, in other words, the shadow of a crescent), with lower angles leading to more narrow crescents.^25,46^ This angle was kept constant at 30°. The azimuthal angle *γ* controls the contour length, since it determines the overlap between the two constituting gold evaporations steps and therefore controls the area at the tips that is selectively functionalized.

### Tuneability of nanocrescents and impact of passivation

The optical response of plasmonic nanocrescents crucially depends on the resonator geometry and the state of polarization.^24,25,44,45^ The fundamental dipole resonance is excited along the long axis, and historically termed the C1 mode as the crescents reads as the letter “C” when the electric field is vertical. Accordingly, the second higher order mode is called C2. Light polarized along the short axis exhibits the first order resonance, historically denoted as U1, as the nanocrescent reads as the letter “U” if the electric field is oriented vertically.^24,25^ For a given colloidal particle mask, we can tune the resonance of an array of combined nanocrescents by varying the azimuthal angle *γ* between the two evaporation steps as previously reported.^25^

**Fig. 3:**
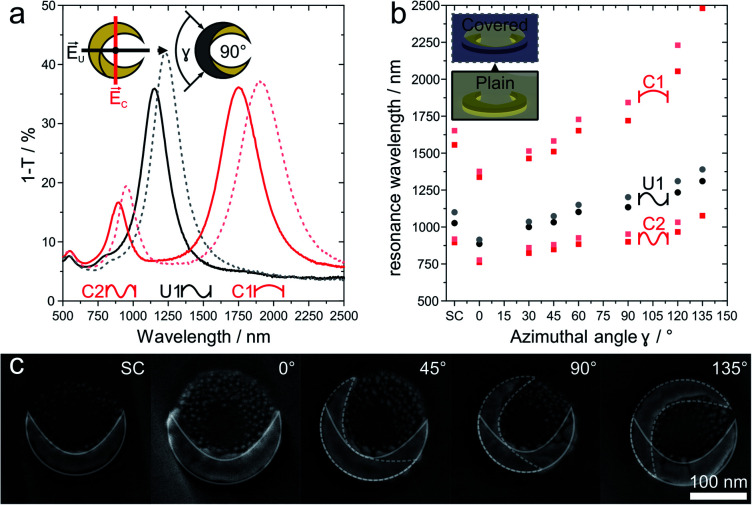
Optical properties response of combined and passivated nanocrescents with different azimuthal angles *γ*. (a) In the case of combined nanocrescent with *γ* = 90° (solid line), linearly polarized light along the long-axis excites the fundamental resonance (C1) at around 1720 nm and the second-order resonance (C2) at around 900 nm. Perpendicularly polarized light excites the first-order resonance (U1) at around 1134 nm. The addition of a passivation layer (5 nm titanium, plasma oxidized to titania) leads to redshifts for all three resonances (dashed lines). (b) The resonance behavior of an array of combined nanocrescents produced from the same colloidal mask can simply be tuned by varying the azimuthal angle *γ* between the evaporation steps. Increasing azimuthal angles increase the contour and leads to a redshift of the resonances (dark filled). The presence of the passivation layer is evidenced by a further redshift of all resonances (shaded filled). (c) High magnification SEM images show the single and the combined nanocrescents made from the same colloidal mask with increasing azimuthal angle *γ* varying from 0° to 135°. The contour of the individual deposition steps is outlined in white color for clarity. More SEM images can be found in Fig. S1.†

Fig. 3 summarizes the response of such combined nanocrescent arrays formed from identical silica colloidal particles used as mask.^43^ With an increasing angle *γ*, the contour length of the nanocrescent resonator increases, leading to a redshift of all three excited resonances. We investigate the influence of the passivation layer deposited on the center of the nanocrescent by removing parts of the samples after the reaction ion etching step (Fig. 2iii), while modifying the rest with the passivation layer. The addition of the dielectric passivation layer (Fig. 3b) redshifts the resonant wavelengths further,^47^ and is in good agreement with previous studies that utilized nanocrescents for dielectric sensing, indicating that the titanium layer was complete converted to insulating titania.^35,37,38^ The red-shifts are in the order of 2.0 to 8.2% and correlate with the azimuthal angle (Fig. S2†) for this thin 5 nm film.

### Site-specific functionalization of arrays of nanocrescents

We demonstrate and verify the fidelity of the site-selective functionalization by functionalizing the tip regions of the nanocrescents with gold nanoparticles of a diameter of approx. 12 nm (AuNPs). We use a positively-charged cysteamine thiol self-assembled monolayer to functionalize the tip region of the nanocrescents and electrostatically bind negatively charged AuNPs, as schematically outlined in Fig. 2. As the gold particles are visible in electron microscopy, we can visualize and quantify the functionalization.

**Fig. 4:**
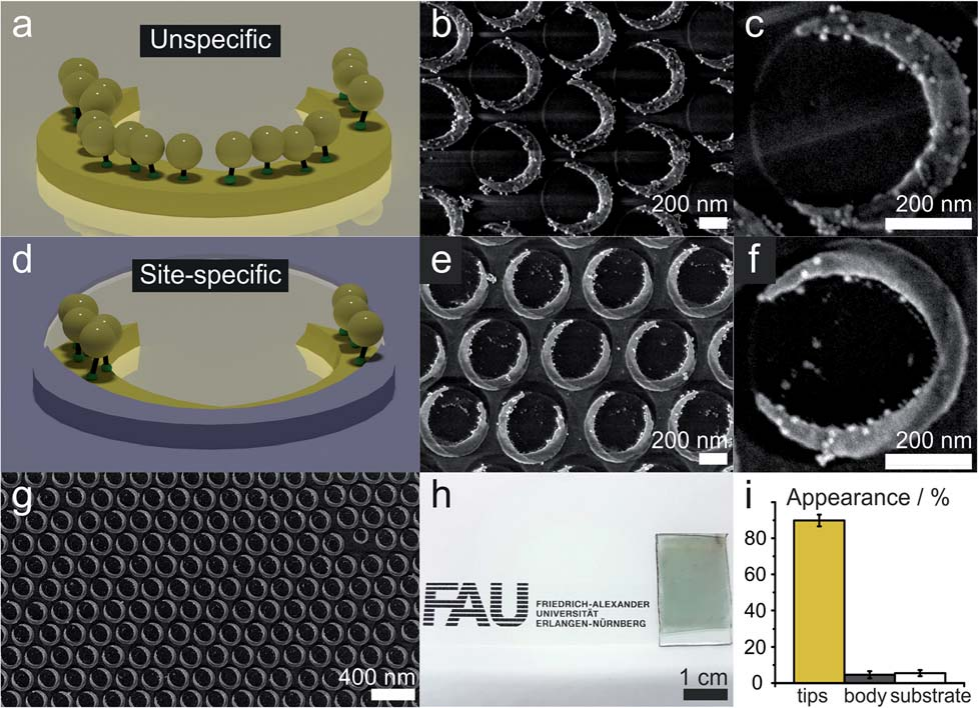
Site-specific functionalization of arrays of gold nanocrescents. The gold nanocrescents were functionalized with positively charged cysteamine self-assembled monolayers and negatively charged gold nanoparticles (AuNPs) were electrostatically adsorbed. (a–c) Functionalization of conventional nanocrescents leads to an even distribution of the AuNPs on the crescent area. (d–f) Site-specific functionalization on the passivated nanocrescents selectively binds AuNPs onto the region of the nanocrescents. The titania passivation layer can be seen by its darker shade in the SEM images. (g and h) The colloidal lithography process enables the fabrication of accurate nanostructures over macroscopic areas. (i) Statistical analysis of the AuNP deposition of the site-specifically functionalized sample. Around 89.9% of the AuNPs were deposited in the predefined areas, while only 4.6% appeared on the covered central area of the nanocrescent and 5.5% were found on the substrate.

Without a passivation layer, the AuNP uniformly coat the cysteamine-functionalized nanocrescents (Fig. 4a–c), demonstrating that the electrostatic adsorption process is efficient to deposit nanoparticles onto the surface nanostructure.

In contrast, the site-specific functionalization of the passivated nanocrescent show gold nanoparticles only at the tip region (Fig. 4d–g). The titania passivation layer can be clearly distinguished by its lower brightness in the high magnification SEM image in Fig. 4e and a predominant deposition of the gold nanoparticles in the uncovered area is seen in Fig. 4f. Fig. 4g and h show the high accuracy of the colloidal lithography process that is capable of creating macroscopic areas of surface nanostructures with high accuracy.

To quantify the accuracy of our selective functionalization method, we statistically analyzed the localized functionalization by determining the number and position of AuNPs from ten SEM images randomly taken from different substrate regions of a sample with an azimuthal angle of 135°. Around 440 nanocrescents and 6300 AuNPs were considered in this procedure (details in Fig. S3†). In the chosen sample, the free accessible gold area at the tips accounts for only around 13% of the total surface area. The statistical analysis revealed that 89.9% of all AuNPs were deposited at these predefined tip region, while only about 4.6% and 5.5% appeared on the covered part of the nanocrescent and the substrate, respectively, which makes about 87% of the total surface area (Fig. 4i).

### Transfer the approach to different geometries

Having demonstrated the general feasibility of the site-specific functionalization by modification of the colloidal lithography process, we transfer the concept to other nanostructures to highlight the versatility of the method. Besides the chosen examples, the methodology could be applied to other geometries produced by colloidal lithography such as geometries with nano-gaps,^48,49^ complex, lattice depended shadow sphere lithography,^50^ nano-hole lithography,^51,52^ nano-imprint lithography,^53^ moiré lithography,^54^ or more conventional lithography methods.

**Fig. 5:**
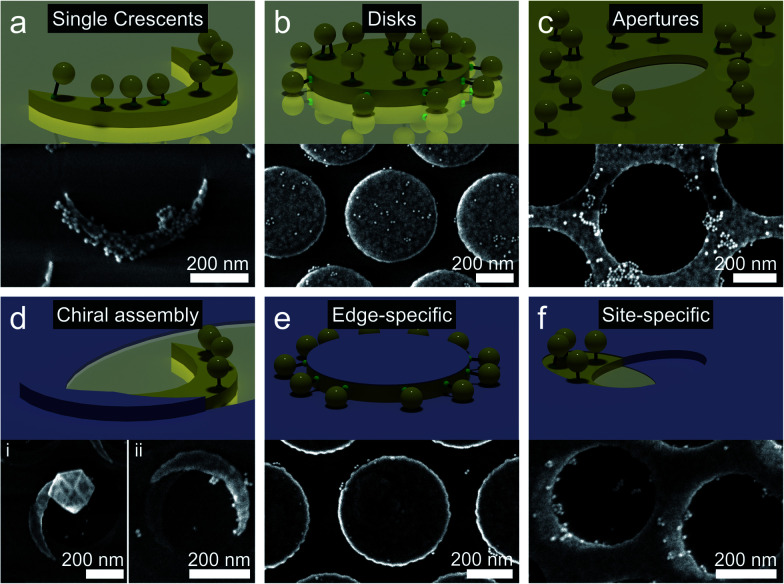
Site-specific functionalization of different surface nanostructures made by colloidal lithography. (a–c) Unspecific functionalization with thiols uniformly deposits gold nanoparticles onto the entire surface of the nanostructure, exemplarily demonstrated for (a) single nanocrescents, (b) nanodisks, and (c) nanoholes. (d–f) Modification of the fabrication process by adding a passivation layer under a different deposition angle selectively uncovers specific parts of the nanostructure and provides a tool to direct the deposition of the nanoparticles onto predefined areas. (d) Rotated evaporation of the passivation layer selectively blocks one tip of a nanocrescent, enabling the formation of structures with a chiral character upon selective adsorption of nanoparticles. (e) Direct evaporation of the passivation layer onto nanodisks blocks the top surface and allows selective functionalization of the edge of the disc. (f) Azimuthal rotation of the passivation layer on top of a gold nanohole arrays enables an asymmetric functionalization of the array with AuNPs.

Fig. 5a–c shows the functionalization of conventional single nanocrescents, nanodisks and nanohole arrays with gold nanoparticles using the same thiol chemistry as before. As expected, a uniform coverage throughout the entire surface of the nanostructures results. Fig. 5d–f shows examples of modification procedures of the colloidal lithography process to afford site-selective functionalization of the same structural motifs. A single tip of a simple nanocrescent can be covered by evaporating of the passivation layer with a rotated evaporation angle (90° in the example). Subsequent functionalization with positively charged thiols and differently-sized AuNPs produces nanostructures with geometric chirality (Fig. 5d), which is interesting in the context of large area manufacturing of arrays of chiral nanocrescents.^55,56^ Covering the top of the nanodisks with a passivation layer provides structures that can be selectively functionalized on their edges (Fig. 5e).

Gold nanohole arrays can be passivated by evaporation of the titanium layer at a different angle, allowing the placement of particles with controlled asymmetries (Fig. 5f).

The examples of Fig. 5 briefly show the potential of selective passivation for a few examples. Noteworthily, colloidal lithography processes have been developed to produces a great wealth of different structural motifs,^50^ which can be subjected to similar process modifications to produce more complex, hybrid nanostructures by secondary assembly of particles, either *via* convenient electrostatic attraction,^58^ or using more sophisticated methods such as DNA origami.^57–60^

## Conclusions

In this manuscript, we demonstrated a reliable, yet simple method to selectively address the tips of nanocrescents, where their near-field enhancement is maximized. The method is based on colloidal lithography and thus able to produce macroscopic arrays of selectively functionalized nanostructures with high accuracy without requiring sophisticated instrumentation or chemistry.

The introduced method provides several degrees of freedom to tailor, for example, the amount and region of the free, accessible gold area. A quantitative evaluation of the selective functionalization showed a high accuracy of gold particles deposited onto the targeted tip region. We quantitatively validated the functionalization by adsorbing oppositely-charged gold nanoparticles, finding a selective adsorption to the functionalized tips. We further show that the concept can be easily transferred to other nanostructure geometries by modification of the colloidal lithography process. The proposed methodology therefore provides a convenient strategy to enhance complexity in the fabrication of nanostructures by enabling the precise decoration of nanostructures with nanoobjects at predefined surface areas and may serve to direct functional molecules to specific sites to enhance efficiency of plasmon-driven applications.

## Experimental part

Experimental part is described in the ESI.†

## Conflicts of interest

The authors declare no competing interests.

## Supplementary Material

NA-002-C9NA00757A-s001
