# Supplementary material for: Addressing the plasmonic hotspot region by site-specific functionalization of nanostructures
Source: Nanoscale Adv. 2019 Dec 4;2(1):394–400. doi: 10.1039/c9na00757a (PMC9418013; doi:10.1039/c9na00757a)
Supplement: NA-002-C9NA00757A-s001 [file NA-002-C9NA00757A-s001.pdf]

Electronic Supplementary Material (ESI) for Nanoscale Advances.
This journal is © The Royal Society of Chemistry 2019

Supporting Information

Addressing the plasmonic hotspot region by site-specific functionalization of nanostructures

*Eric S.A. Goerlitzer+, Lutz E. Speichermann+, Talha A. Mirza, Reza Mohammadi, Nicolas Vogel**

+ Authors contributed equally to this work

** Corresponding author*

Experimental part

Fabrication of evaporation masks

Monodisperse polystyrene particles were synthesized by surfactant-free emulsion polymerization.1 The dispersion was cleaned to remove surface active impurities that can interfere with the assembly process, following a protocol from literature.2 Microscope slide substrates (76 x 26 x 1 mm³, Carl Roth) were cut in smaller pieces (approx. 15,2 x 26 mm²) and cleaned using EtOH (denaturated, 96 %, Carl Roth) and oxygen plasma (Femto SLS, Diener electronic GmbH + Co. KG, 4 sccm O2, 100 W, 5 min.). The assembly of monolayer of polymer beads at the air-water interface followed an established protocol.3 Non-closed packed colloidal monolayers were fabricated by shrinking the polymer particles using the same plasma oven at reduced power.4 The direct assembly of non-closed packed silica particles was performed using SiO2@PNIPAm core-shell particles assembled at the air/water interface of a Langmuir Blodgett trough as described elsewhere.5 The latter method was used for the results in Figure 3, i.e. when spectra between different azimuthal angles were compared.

Fabrication of nanocrescents

Adhesion layer (1 nm titanium, ≥ 99.6 %, granules, HMW Hauner) and gold (20 nm, ≥ 99.99 %, granules, HMW Hauner) were consecutively deposited using a thermal evaporator (THE3-2KW, Torr International Inc.), with the substrate lifted at an elevation angle α = 30°. The second gold layer (20 nm) was evaporated by rotating the samples manually to the desired azimuthal angle γ before the deposition process. Next, combined nanocrescents were produced by reactive ion etching along the surface normal using a directed argon ion etching (100 W, PlasmaPro NGP 80, Oxford Instruments). To produce the site-specific covered combined nanocrescents (CCC) the samples were turned to half the azimuthal angle γ in the metal evaporator. A thin layer of 5 nm titanium was evaporated as a passivation layer. Finally, the colloidal particles used as mask were removed using adhesive tape (Scotch Tape, 3M). The titanium was oxidized to titania using oxygen plasma treatment (10 min, 4 sccm O_2, 100W, Diener, Model Femto). Other geometries used same thicknesses and evaporation angles as described in the text.

Functionalization of gold nanostructures

Substrates were cleaned by successive immersion in THF, Acetone, Isopropanol/EtOH (1:1) and ultrapure water (MilliQ, 18 MΩ) followed by gentle drying with nitrogen. To remove organic residuals, ensure the oxidation of the passivation layer and improve surface wettability, oxygen plasma was applied as described above. The substrates were then functionalized by submersion in a cysteamine hydrochloride (98.0%, Sigma Aldrich) EtOH (absolute, 99.5%) solution (20 mmol) overnight. Excess amounts were removed by extensive rinsing with EtOH (absolute, 99.5%). Gold nano particles were prepared using the Turkevich method and stored in a fridge.[6] The citrate molecules acting as electrostatic stabilizers were replaced with 6-mercaptohexanoic acid (90%, Sigma Aldrich) as follows. One part of aqueous thiol solution (20 mmol, MilliQ, 18 MΩ, pH ~ 4) was added dropwise to five parts particle dispersion (as synthesized) and another five parts ultrapure water (MilliQ, 18 MΩ) to prevent aggregation. Then, the mixture was stirred for 1-2 h to achieve proper surface coverage. As a last step, the functionalized substrates were submerged in the gold nanoparticle dispersion for at least 2 h. Excess particles were removed by rinsing with ultrapure water (MilliQ, 18 MΩ).

Characterization

SEM images were taken with a GeminiSEM 500, Zeiss. UV-Vis-NIR spectra were measured using a conventional spectrometer (Lambda 950, Perkin-Elmer) with a Glan Thompson prisma polarizer drive in transmission mode. Illumination areas were about 7 x 9 mm².

Further Information

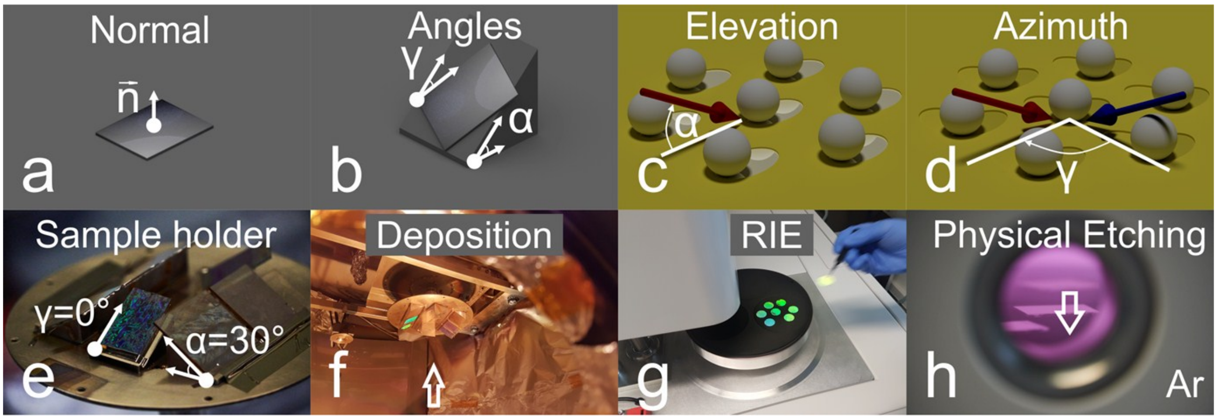

Figure S1. Geometry and parameter definitions for the colloidal lithography method to produce site-specific functionalized combined crescents. a) The surface normal $\vec{n}$ of a substrate. b) Samples are placed on a holder with an elevation angle α of 30° for further evaporation steps. The twisting angle γ is defined as the twisting angle of the substrate on the holder´s surface, where γ = 0° describes a parallel alignment of the longer sides. Positive angles are twisting in a clock-wise direction. c) Due to the before mentioned elevation due to the sample holder, material is being deposited underneath the masking sphere with an ellipsoidal shadow. The deposition underneath is required to render the crescent shaped resonators. d) The azimuthal angle between the two evaporation steps (here γ < 0°) defines the overlap of the material underneath the spheres. e) A typical samples are shown on a α = 30° sample holder with a azimuthal angle of 0° (Note: these are larger polystyrene spheres with diameters above 400 nm). f) The sample holder is put upside down in our vacuum chamber, where the evaporation sources are located at the bottom. g) The directed physical etching is carried out in a conventional reactive ion etcher, with the surface normal showing upwards (flat). h) Argon ions are milling away all material layers that are not shielded by the particels´shadows.

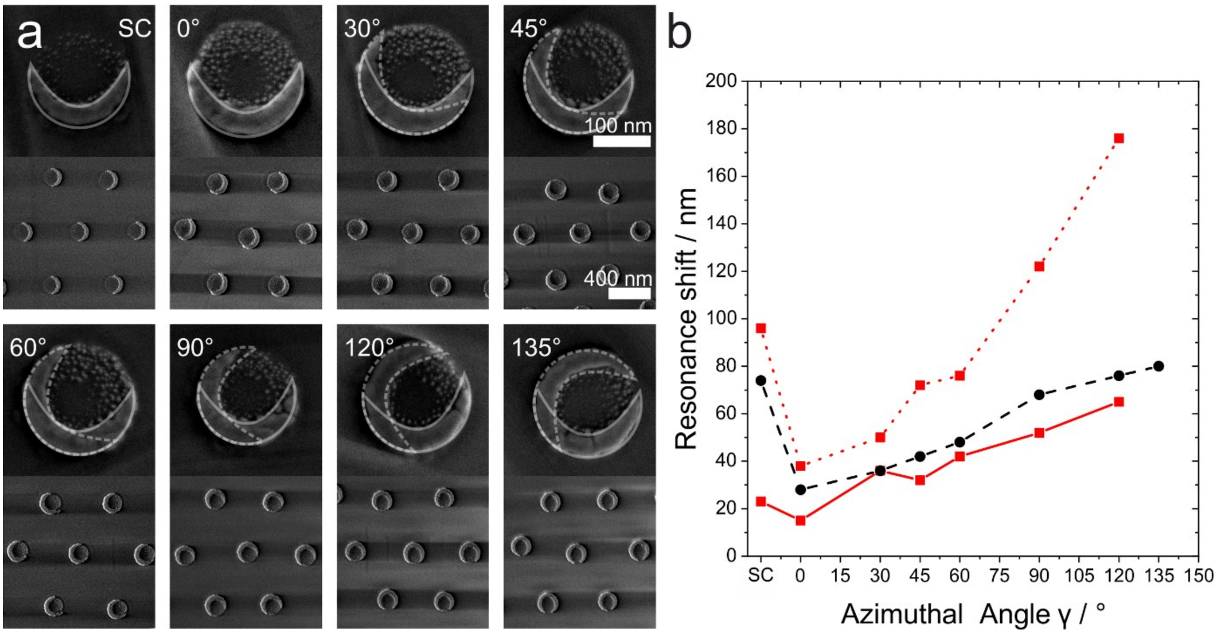

Figure S2. Combined and passivated crescents with different azimuthal angles γ. a) SEM images of higher and lower magnification of a single crescent and combined crescents with γ ranging from 0° (i.e. second gold layer directly on top) to 135°. b) Addition of a passivating titania layer at γ/2 leads to red-shifts. The shift scales with the azimuthal angle for combined crescents.

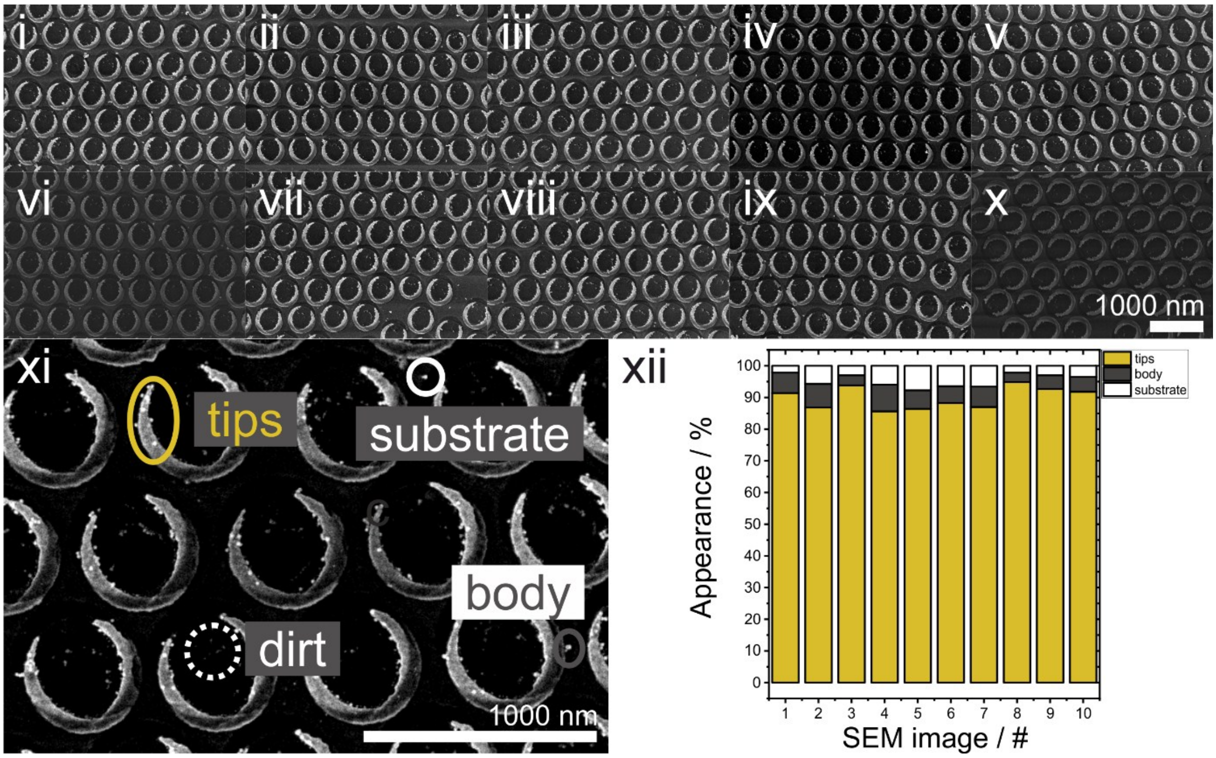

Figure S3. Images of the statistical analysis of site-specific functionalized combined crescents. Images were taken from a typical sample at different location all over the substrate. i-x) A total of 447 crescents were considered, and 6283 gold nano particles were manual tracked. We found an average appearance of the nanoparticles of: 89.9% ± 3.2% at the tips of the crescents, 5.5% ± 1.72% at the substrate and 4.6% ± 2.0% at the covered body of the crescent. The site-specific functionalized tips make around 13% of the total area, while covered body and substrate make the remaining 83%. xi) The appearances of the nanoparticles were counted manually with the aid of ImageJ, were we considered three different areas, tips (bright appearance), the main body (dark appearance) and the remaining substrate. Xii) Statistical appearance of nanoparticles at the different regions for for the 10 SEM images considered above. Note that due to the process, the inner wall of the nanostructures is not covered by the titania passivation layer. Due to its small height, the efficient attachment of AuNPs seems hindered, resulting in only a very small number of particles attached to the inner wall of the crescent.

References

1 J. W. Goodwin, J. Hearn, C. C. Ho and R. H. Ottewill, *Colloid Polym. Sci.*, 1974, **252**, 464–471.

2 M. Rey, T. Yu, R. Guenther, K. Bley and N. Vogel, *Langmuir*, 2019, **35**, 95–103.

3 N. Vogel, S. Goerres, K. Landfester and C. K. Weiss, *Macromol. Chem. Phys.*, 2011, **212**, 1719–1734.

4 M. Retsch, M. Tamm, N. Bocchio, N. Horn, R. Förch, U. Jonas and M. Kreiter, *Small*, 2009, **5**, 2105–2110.

5 J. S. J. Tang, R. S. Bader, E. S. A. Goerlitzer, J. F. Wendisch, G. R. Bourret, M. Rey and N. Vogel, *ACS Omega*, 2018, **3**, 12089–12098.

6 J. Turkevich, P. C. Stevenson and J. Hillier, *Discuss. Faraday Soc.*, 1951, **11**, 55.
